# The Gene Expression Analysis of Peripheral Blood Monocytes From Psoriasis Vulgaris Patients With Different Traditional Chinese Medicine Syndromes

**DOI:** 10.3389/fphar.2021.759741

**Published:** 2022-01-19

**Authors:** Yue Lu, Yao Qi, Li Li, Yuhong Yan, Jianan Wei, Danni Yao, Jingjing Wu, Hao Deng, Jingwen Deng, Shuyan Ye, Haiming Chen, Qubo Chen, Hengjun Gao, Ling Han, Chuanjian Lu

**Affiliations:** ^1^ State Key Laboratory of Dampness Syndrome of Chinese Medicine, the Second Affiliated Hospital of Guangzhou University of Chinese Medicine (Guangdong Provincial Hospital of Chinese Medicine), Guangzhou, China; ^2^ Guangdong Provincial Key Laboratory of Clinical Research on Traditional Chinese Medicine Syndrome, Guangzhou, China; ^3^ Guangdong-Hong Kong-Macau Joint Lab on Chinese Medicine and Immune Disease Research, Guangzhou University of Chinese Medicine, Guangzhou, China; ^4^ Shanghai Molecular Medicine Engineering Technology Research Center, Shanghai, China; ^5^ Shanghai National Engineering Research Center of Biochip, Shanghai, China

**Keywords:** gene chip, gene expression, psoriasis vulgaris, TCM syndrome type, peripheral blood monocytes

## Abstract

Psoriasis is chronic skin disease and an important health concern. Traditional Chinese Medicine (TCM) has shown great promise in the treatment of psoriasis. However, the correlation between TCM Syndromes and genomics of psoriasis has not been evaluated. Here, we analyzed gene expression profiling of monocytes from psoriasis vulgaris patients with different TCM syndrome types to reveal the molecular basis of different psoriasis syndromes. Of the 62 cases of psoriasis vulgaris recruited, 16, 23, and 23 cases were of blood-heat syndrome, blood stasis syndrome, and blood-dryness syndrome, respectively; 10 healthy controls were recruited as controls. Affymertix’s Gene Chip ®clariom D gene chip was used to detect the gene expression profile of peripheral blood monocytes collected from recruited individuals. Compared with the healthy control group, 1570 genes were up-regulated and 977 genes were down-regulated in the psoriasis vulgaris patients group; 798 genes and 108 genes were up- and down-regulated in the blood-heat syndrome group respectively; 319 and 433 genes were up- and down-regulated in the blood-dryness syndrome group, respectively; and 502 and 179 genes were up-and down-regulated in the blood-stasis syndrome group. Our analyses indicated not only common differential genes and pathways between psoriasis syndrome groups and healthy controls, but also syndrome-specific genes and pathways. The results of this study link the three syndromes at the gene level and will be useful for clarifying the molecular basis of TCM syndromes of psoriasis.

**Clinical Trial Registration:** (http://www.chictr.org.cn/showproj.aspx?proj=4390), identifier (ChiCTR-TRC-14005185).

## Introduction

Psoriasis is a chronic proliferative skin disease mediated by abnormal immune system, which is determined by polygenic inheritance and stimulated by multiple environmental factors. Globally, 2–3% of the population is affected by psoriasis; currently, there are approximately 125 million psoriasis patients in the world. In China, the total incidence rate is 0.72%, with the current estimation being 10 million patients (Langley et al., 2005; Menter et al., 2008; Perera et al., 2012). Traditional Chinese Medicine (TCM) has great advantages and broad prospects in the treatment of psoriasis, and has accumulated rich treatment experience over a long time (Yu et al., 2013; Zhang et al., 2014; Zhang et al., 2016; Yu et al., 2017). PSORI-CM02 and FZHFZY has been used for decades to treat blood stasis and blood dryness syndromes of psoriasis vulgaris at the Guangdong Provincial Hospital of Chinese Medicine (Lu et al., 2019; Lu et al., 2021a; Lu et al., 2021b). PSORI-CM02 consists of Rhizoma Curcumae (*Curcuma longa* L.), Radix Paeoniae rubra (*Paeonia lactiflora* Pall.), *Sarcandra glabra* [*Sarcandra glabra* (Thunb.) Nakai], Rhizoma Smilacis glabrae (*Smilax glabra* Roxb.), and Fructus mume [*Prunus mume* (Siebold) Siebold & Zucc.] (the ratio was 2: 3: 5: 5: 2). FZHFZY includes Radix seu Herba Cynanchi Paniculati (*Vincetoxicum mukdenense* Kitag*.*), Densefruit Pittany Root-bark (*Dictamnus dasycarpus* Turcz.), Fructus Cnidii [*Cnidium monnieri* (L.) Cusson], Rhizoma Smilacis Glabrae (*Smilax glabra* Roxb.), Radix Rehmanniae Preparata [*Rehmannia glutinosa* (Gaertn.) DC.], Radix Angelicae Sinensis [*Angelica sinensis* (Oliv.) Diels], and Pericarpium Punicae Granati (*Punica granatum* L.) (the ratio was 3: 3: 2: 3: 3: 2: 3).

We have analyzed the distribution of syndromes in psoriasis vulgaris cases from 1979 to 2010 reported in literature, and found that among all syndromes in these patients, the top three syndromes, namely blood heat, blood dryness, and blood stasis syndromes accounted for 75.85%. Moreover, blood stasis syndrome was related to the stable phase, while blood dryness syndrome was related to regression phase (Lu CJ. et al., 2012; Yan et al., 2012). At present, there is no research on the correlation between TCM syndromes and genomics of psoriasis. Therefore, we carried out gene expression profiling to analyze the expression levels of genes associated with blood heat, blood dryness, and blood stasis syndromes, which are common TCM syndromes of psoriasis. We further examined the differences in gene expression among the three syndromes in order to reveal the molecular basis of the different syndromes of psoriasis at the gene level.

## Materials and Methods

### Patient Recruitment and Characteristics

All recruited patients in this study were in the clinic and wards of the Department of Dermatology of Guangdong Provincial Hospital of Chinese Medicine. This study was approved by the Ethics Committee of our hospital, and all patients and healthy volunteers provided informed signed consent. The psoriasis vulgaris group comprised of 62 cases in total, including 16 cases of blood heat syndrome, 23 cases of blood stasis syndrome, and 23 cases of blood dryness syndrome. Of these, 44 were males and 18 were females; the average age was 45.5 years (range, 19–60 years). The disease course was 1 month–10 years. The diagnostic criteria were in line with those described for psoriasis vulgaris (Boehncke and Schön, 2015); the TCM diagnosis was consistent with that of *bai bi* (TCM disease name for psoriasis) (Lu C. J. et al., 2012). The diagnostic criteria of TCM syndrome types refer to the local standard of Guangdong Province–Psoriasis vulgar syndrome differentiation standard of traditional Chinese medicine (2018 Edition), as follows. Blood heat syndrome: in the progressive stage, the skin lesions are bright red, consciously hot, dry mouth and like drinking, upset and irritable, dry stool, yellow urine, red tongue or yellow tongue coating, and rolling and rapid pulse; blood stasis syndrome: in the static stage, the skin lesions are dark red or purple in color, thick lesion plaques, obvious infiltration, the scales are attached tightly, hard to peel off, dark purple tongue or ecchymosis, ecchymosis, astringent or slow pulse; blood dryness syndrome: in the static or regression stage, the skin color is light red, consciously dry, with many scales and easy to fall off, conscious fatigue, dry mouth and throat, light tongue, little or no moss, and the pulse is heavy or slow. The healthy control group comprised of 10 individuals that were proven to be healthy by examinations at the outpatient clinic; their average gender and age distributions matched that of the psoriasis group.

### Exclusion Criteria

Pregnant or lactating women, those orally administered with steroid drugs and/or retinoic acids or biological agents within the past 6 months, those given topical application of steroid preparations or retinoic acid cream within the past 1 month, patients with hyperthyroidism, cardiovascular diseases, cerebrovascular diseases, liver diseases, kidney diseases, hematopoietic system diseases and other primary diseases, patients with mental diseases, patients on long-term medications, and patients with mixed TCM syndromes were all excluded.

### Instruments and Reagents

The TRI Reagent (Sigma, Germany, Item No. T9424), miRNeasy Micro Kit (QIAGEN, Germany, Item No. 217084), RNase-Free DNase Set (QIAGEN, Germany, Item No. 79254), Affymetrix gene expression profiling kit GeneChip® WT PLUS Reagent Kit (Affymetrix, US, Item No. 902280), GeneChip® Hybridization, Wash and Stain Kit (Affymetrix, US, Item No. 900720), shaking hybridization oven (Affymetrix, US, Item No. 00-0331-220 V), washing workstation 450DX2 [Affymetrix, US, Command Console Software 3.1 (Affymetrix, Santa Clara, CA, US), Item No. 00-0335], data acquisition software Command Console Software 3.1 (Affymetrix, US), data analysis software Transcriptome Analysis Console Software (Affymetrix, US); Agilent Bioanalyzer 2100 Electrophoresis Instrument (Agilent, US); chip scanner GeneChip^®^ Scanner 3000DX2 (Affymetrix, US, Item No. 00-0334); and GeneChip^®^ Clariom D (Affymertix, US) were obtained from the indicated manufacturers.

### Sample Collection and Processing

Venous blood (10 ml) was collected from the median cubital vein into an anticoagulant tube containing heparin. Peripheral blood monocytes (PBMC) were isolated by Ficoll density gradient centrifugation method (Mendez-David et al., 2013) with lymphocyte separation solution (absin, China, item No. abs930b). First, suck the lymphocyte layer to a clean centrifuge tube and add 6 ml PBS to it, gently blow to fully mix the lymphocyte layer with PBS, then centrifuge the centrifuge tube at 60–100 g at 18–20°C for 10 min. Remove the upper separation solution, add 6–8 ml PBS to the centrifuge tube, gently blow with a Pasteur pipette to fully mix the lymphocytes, centrifuge for 10 min at 18–20°C and 100 × g, and remove the upper separation solution to obtain PBMC and stored in a refrigerator at −80°C.

### RNA Extraction From Samples

The total RNA was extracted from the samples using the TRI Reagent according to the manufacturer’s instructions. Then, miRNeasy Micro kit and RNase-Free DNase Set were used to purify total RNA. After examining the quality, the purified total RNA was evaluated for RNA integrity using the Agilent Bioanalyzer 2100 electrophoresis apparatus.

### *In vitro* Reverse Transcription Amplification and Labeling of RNA Samples

Affymetrix gene expression profiling kit GeneChip® WT PLUS Reagent Kit was used to carry out *in vitro* reverse transcription amplification of the mRNA from the samples according to the manufacturer’s instructions. At the same time, biotin was used to label cRNA.

### Chip Hybridization and Elution

According to Affymetrix’s chip hybridization procedure, the biotin-labeled cRNA was added to the cartridge gene chip, the matching reagent kit was used, and the chip was subjected to hybridization for 16 h in the shaking hybridization oven at 45°C. After hybridization was completed, the chip was washed in the washing workstation according to the standard operating procedure.

### Chip Scanning and Data Processing

The chip results were scanned using GeneChip® Scanner 3000DX2, the raw data were read by Command Console Software 3.1, and the data that passed quality control were normalized by Command Console Software 3.1 software. The Gene level_SST_RMA algorithm was used to perform quality control on the data and issue the data report, and the Transcriptome Analysis Console software was used to perform preliminary analysis on gene differential expression. The differentially expressed genes with *p* < 0.05 and fold change ≥2 were screened out and enriched using DAVID, and the pathways with *p* < 0.05 were selected for analysis. To determine the biological functions or signalling pathways affected by the differentially expressed genes, the Kyoto Encyclopedia of Genes and Genomes (KEGG) enrichment analysis was performed using the online metascape tool (https://metascape.org/gp/index.html#/main/step1).

### Validation by Quantitative Real-Time Polymerase Chain Reaction

The isolated RNA from the PBMC samples from different groups was reverse transcribed directly into cDNA using a SuperScript IV Reverse Transcriptase (Thermo Fisher, Dalian, China) according to the manufacturer’s instructions. PCR amplification conditions were as follows: initial denaturation at 95°C for 30 s, followed by 40 cycles of denaturation at 95°C for 5 s and 60°C for 30 s, then annealing at 95°C for 5 s and 60°C for 1 min. *USP17L15*, *JUND*, *OR1J1*, *FAM98B*, *HNRNPA2B1*, *NDUFA6*, *NDUFS8*, *UQCRC1*, *FAM208A*, *METTL15*, *USP17L19*, *CTLA4*, *HEMGN*, *SLC24A4*, *SLC30A6*, *CD9*, *S100A6*, *ITGB3*, *UPF3B,* and *MRPS27* gene expression were examined using qRT-PCR by a SYBR Green PCR kit (TaKaRa, Dalian, China).

### Statistical Analysis

Statistical analyses were performed with SPSS software 19.0 and visualized using GraphPad Prism 5.0 (La Jolla, CA, United States). The results are expressed as means ± standard errors of the means. Student’s *t*-test was used for the statistical analyses. *p* values <0.05 were considered significant.

## Results

### Comparison of Gene Expression Profiles

Preliminary analysis of the gene expression profiles from psoriasis patients compared with those from the healthy control group showed that 1570 and 977 differentially expressed genes were up-regulated and down-regulated, respectively. Comparison of the psoriasis blood heat syndrome group with the healthy control group showed that 798 and 108 differentially expressed genes were up-regulated and down-regulated, respectively. In case of the psoriasis blood dryness syndrome group, 319 and 433 differentially expressed genes were up-regulated and down-regulated, respectively, compared with the healthy control group. Comparing the gene expression profiles from the psoriasis blood stasis syndrome group with those from the healthy control group, we screened out 502 up-regulated and 179 down-regulated differentially expressed genes (Table 1).

**TABLE 1:** Analysis of differentially expressed genes between groups.

	Sample size	Differential genes	Up-regulated genes	Down-regulated genes
The psoriasis patients group vs. the healthy control group	62 vs. 10	2547	1570	977
The psoriasis blood heat syndrome group vs. the healthy control group	16 vs. 10	906	798	108
The psoriasis blood dryness syndrome group vs. the healthy control group	23 vs. 10	752	319	433
The psoriasis blood stasis syndrome group vs. the healthy control group	23 vs. 10	681	502	179

### Principal Component Analysis of the Three Syndromes

**FIGURE 1:**
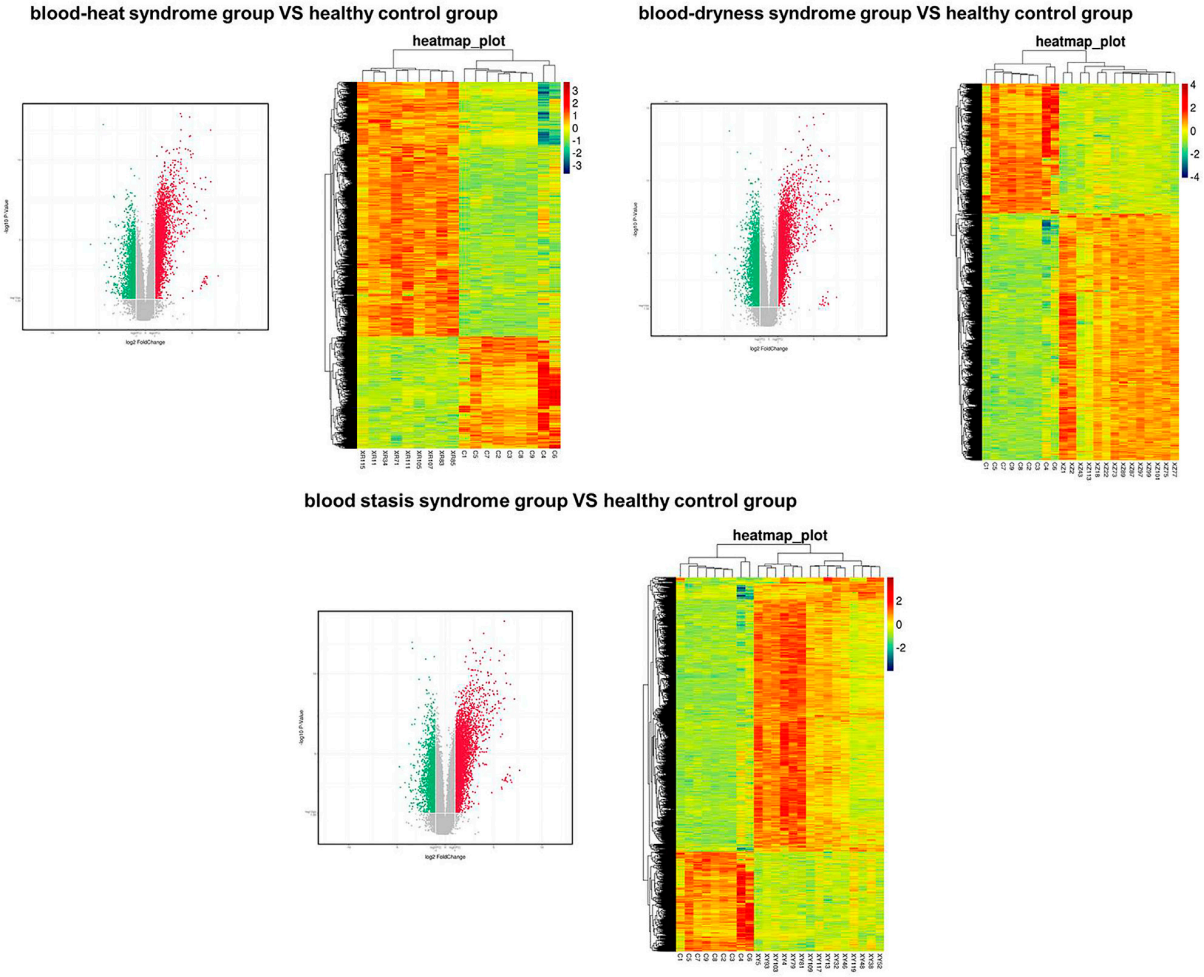
Volcanic map (left panel) and heat map (right) showed the distributions of genes in different syndromes.

**FIGURE 2:**
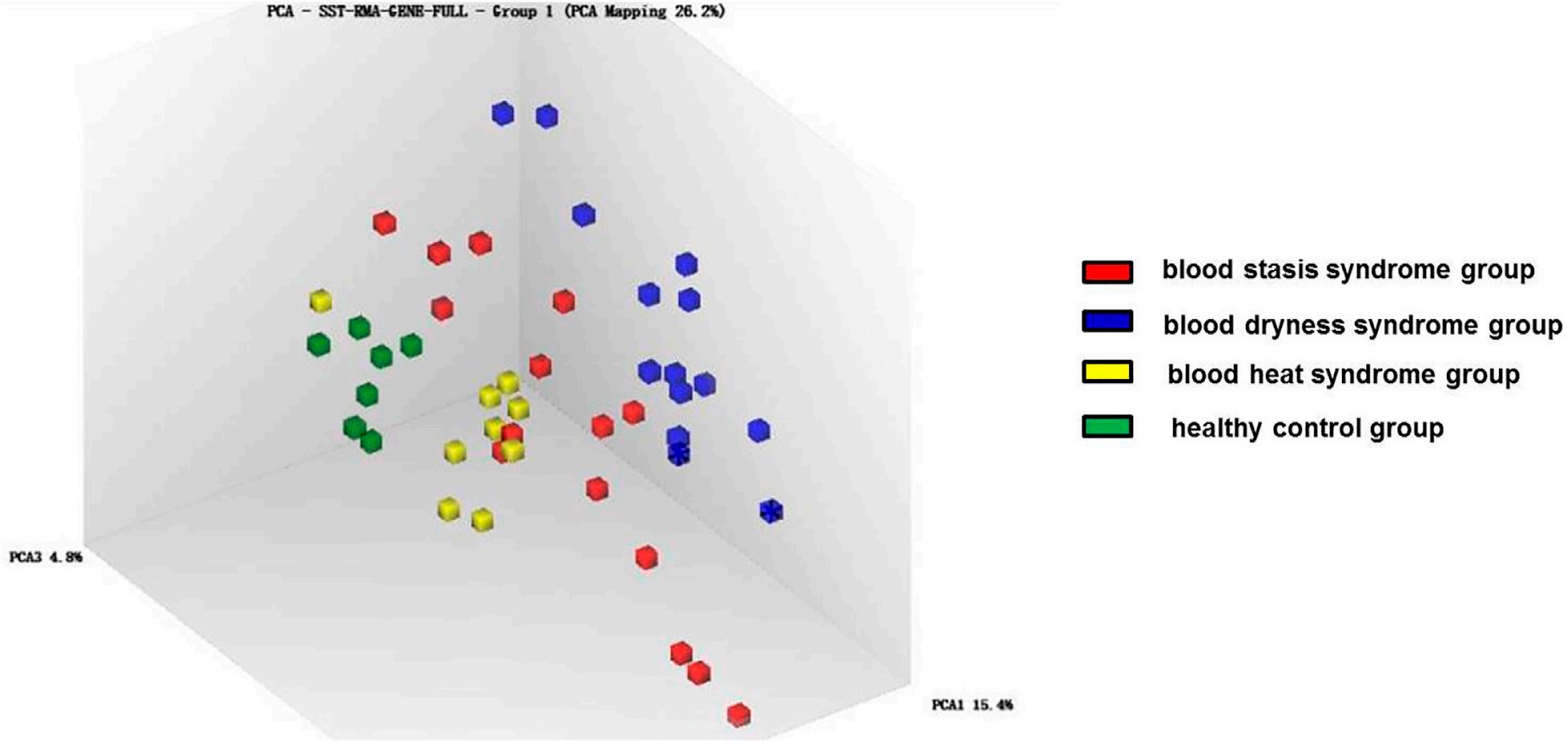
Principal component analysis of gene expression in different syndromes.

Principal component analysis was carried out on the expression profile chip data of the three syndromes of psoriasis, i.e., blood heat, blood dryness, and blood stasis, and it was found that the three syndromes could be clearly distinguished (Figures 1, 2). This indicates that the TCM syndrome differentiation theory of psoriasis is consistent with the differences in gene expression levels among the syndromes.

### Analysis of Differential Genes and Signalling Pathways Between the Three Syndromes and the Control Group

**FIGURE 3:**
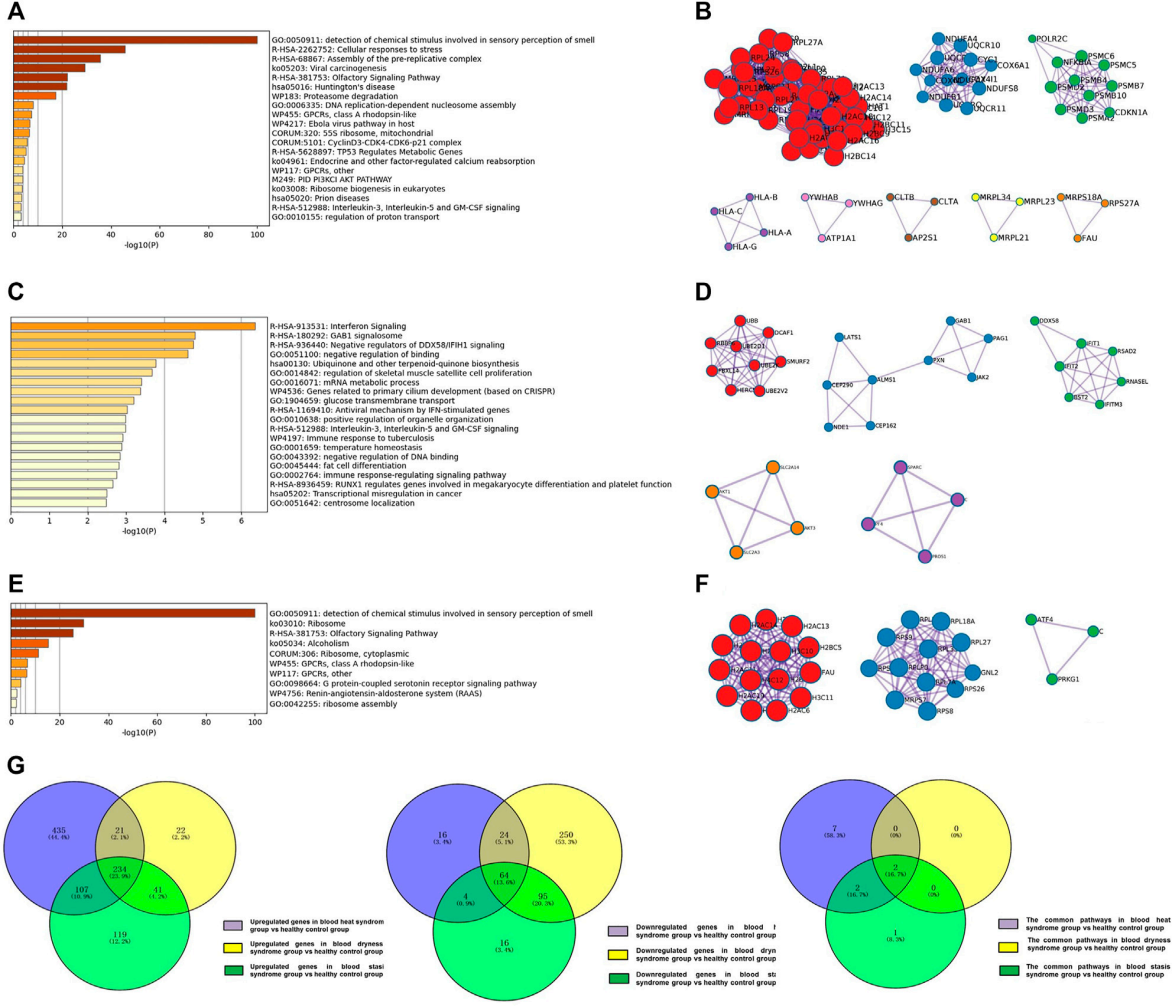
Analysis of regulated differential genes and pathways between three syndromes of psoriasis and healthy control group. **(A)** Analysis of regulated differential pathways between the psoriasis blood heat syndrome group and the control group; **(B)** Analysis of differentially expressed genes between the psoriasis blood heat syndrome group and the control groups; **(C)** Analysis of regulated differential pathways between the psoriasis blood dryness group and the control group; **(D)** Analysis of differentially expressed genes between the psoriasis blood dryness group and the control groups; **(E)** Analysis of regulated differential pathways between the psoriasis blood stasis syndrome group and the control group; **(F)** Analysis of differentially expressed genes between the psoriasis blood stasis syndrome and the control groups; **(G)** Venn analysis of differentially expressed genes and pathways between three syndromes of psoriasis and healthy control group.

The enrichment analysis showed that the top five major signalling pathways in the psoriasis blood heat syndrome group included detection of chemical stimulus involved in sensory perception of smell, cellular responses to stress, assembly of the pre-replicative complex, viral carcinogenesis, olfactory signaling pathway (Figures 3A,B). The top five major signalling pathways in the psoriasis blood dryness group included interferon signaling, GAB1 signalosome, negative regulators of DDX58/IFIH1 signaling, negative regulation of binding, ubiquinone and other terpenoid-quinone biosynthesis (Figures 3C,D). The top five major signalling pathways in the psoriasis blood stasis syndrome group included detection of chemical stimulus involved in sensory perception of smell, ribosome, olfactory signaling, alcoholism, ribosome, and cytoplasmic (Figures 3E,F). Venn diagram analysis of the differential genes was carried out by comparing the expression profile chip data of the three respective psoriasis syndromes with those of the healthy control group. It was found that the three syndromes showed specific differential genes as well as some common differential genes (Figure 3G).

Venn diagram analysis on the pathways associated with the three syndromes showed that blood heat and blood stasis syndromes were associated with their own specific pathways (Table 2). Specific abnormalities (mainly up-regulated) existed in the proteasome and oxidative phosphorylation pathways in the psoriasis blood heat syndrome group, and mitochondrial related ribosome pathway abnormalities (down-regulated) existed in the psoriasis blood stasis syndrome group. The result of KEGG enrichment indicated that the three psoriasis syndromes showed some common pathways (Table 2), including common abnormalities in ribosome and olfactory transduction.

**TABLE 2:** The common pathways and specific pathways in different syndromes.

The common pathways between the three syndromes	Specific pathways in blood heat syndrome	Specific pathways in blood stasis syndrome
Olfactory transduction	Viral carcinogenesis	Ribosome biogenesis in eukaryotes
Ribosome	Proteasome	
	Huntington disease	
	Cardiac muscle contraction	
	Alzheimer disease	
	Parkinson disease	
	Oxidative phosphorylation	

### Verification of Selected Genes

**FIGURE 4:**
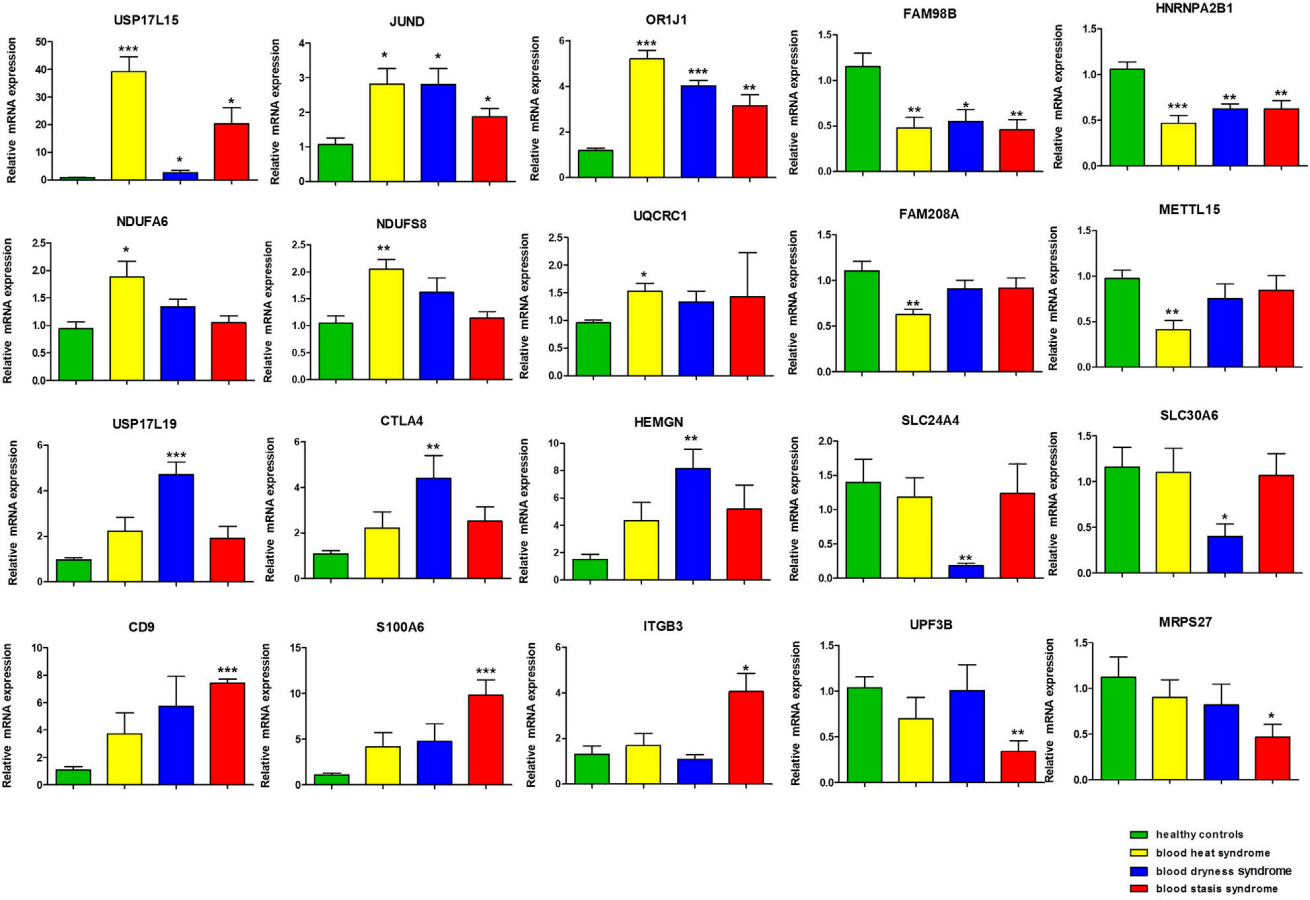
The gene validation by qRT-PCR. (*p < 0.05, **p < 0.01, ***p < 0.001 in qRT-PCR verification when comparing data between different syndrome groups and healthy control group).

Using qRT-PCR, we verified the twenty differentially expressed genes obtained from microarray, which was in accordance with microarray results (Figure 4). The genes *USP17L15*, *OR1J1*, and *JUND* were significantly up-regulated in all three syndromes. *FAM98B* and *HNRNPA2B1* were significantly down-regulated in all three syndromes. In addition, we found that several oxidative phosphorylation pathway-related genes, such as *NDUFS8*, *NDUFA6*, and *UQCRC1*, showed syndrome-specific abnormal up-regulation in the blood heat syndrome group. *FAM208A* and *METTL15* were significantly down-regulated in the blood heat syndrome group. *USP17L19*, *CTLA4*, and *HEMGN* were significantly up-regulated while *SLC24A4* and *SLC30A6* were significantly down-regulated in the blood dryness syndrome group. *CD9*, *S100A6*, and *ITGB3* were significantly up-regulated while *UPF3B* and *MRPS27* were significantly down-regulated in the blood stasis syndrome group.

## Discussion

Psoriasis is a common clinical chronic inflammatory skin disease, and its pathogenesis is still unclear. In this study, the three most common TCM syndromes of psoriasis were analyzed at gene expression level using gene expression profiling chip. There were some common as well as some specific differential genes and pathways between the three TCM psoriasis syndromes and the healthy control group.

In blood heat syndrome, a common syndrome of psoriasis, we found that a large number of oxidative phosphorylation pathway-related genes, such as *NDUFS8*, *NDUFA6*, and *UQCRC1*, showed syndrome-specific expression abnormalities. The literatures showed that these gene abnormalities were associated with mitochondrial metabolic activities (Murray et al., 2003; Angerer et al., 2014; Unni et al., 2019), suggesting a relationship between psoriasis and cellular metabolism. In recent years, epidemiological studies in China and other countries have shown that psoriasis and metabolic syndrome are correlated (Kim et al., 2012; Danielsen et al., 2015). It is generally believed that the common metabolic pathways in the pathogenesis of the two diseases contribute to the correlation. A study has shown that moderate to severe psoriasis often presents clinical features of metabolic disorders (Armstrong et al., 2013).

Ubiquitination is a reversible biological process and participates in the regulation of inflammatory signaling pathways (Farshi et al., 2015; Young et al., 2019). *USP17* is one of the members of the deubiquitinase family. It has been reported that it promotes the growth of lung cancer and the expression of inflammatory genes in cancer (Lu et al., 2018). Our results show that the abnormal up-regulation of *USP17L19* in blood dryness syndrome. It suggested that the induction of inflammation by *USP17* may be involved in the immune dysfunction of psoriasis. Solute carrier (SLC) transporter is a special protein that can transport substrates across cell membranes (Rives et al., 2017). It is responsible for many basic physiological functions, including nutrient absorption, ion transport and waste treatment (Hediger et al., 2004). Some studies believe that it is the gatekeeper of immune cells and an important participant in regulating the metabolic function of immune cells (Song et al., 2020). We found the abnormal expression of solute carrier transporter (*SLC24A4* and *SLC30A6*), which may be an important part of mediating metabolic and immune dysfunction in blood dryness syndrome of psoriasis.

Our result showed that *UPF3B* and *MRPS27* were significantly down-regulated in the blood stasis syndrome group. Mitochondrial Ribosomal protein S27-like (MRPS27) is reported as a physiological regulator of p53, which can suppress genomic instability and tumorigenesis (Xiong et al., 2014). At present, lots of studies on ribosomal proteins are related to cancer, and there is no study related to psoriasis. Ribosomal protein dysfunction is related to cancer, metabolic disorder and inflammation and ribosomal protein ubiquitination is a feedback mechanism of the body (Wang W. et al., 2015; Lin et al., 2021). Our results show that the level of ubiquitinase decreases in psoriasis blood dryness syndrome, while ribosomal protein decreases in blood stasis syndrome, which may suggest that there are some connections and differences between the two syndrome types in inflammation and metabolism.

From the results of pathway analysis, the oxidative phosphorylation pathway-specific abnormalities were associated only with blood heat syndrome. According to literature, skin oxidative phosphorylation, ATP level and ATP enzyme activity level vary in the different stages of psoriasis (Cannavo et al., 2019; Xu et al., 2019). Genetic susceptibility and oxidative stress caused by exogenous and endogenous factors can lead to abnormal differentiation and proliferation of keratinocytes, thus leading to the development and persistence of psoriasis (Zhou et al., 2009; Lin and Huang, 2016). The European S3-Guidelines on the systemic treatment of psoriasis vulgaris--Update 2015 (Nast et al., 2015) clearly point out that skin lesions of psoriasis patients show a lack of cyclic adenosine monophosphate (cAMP), which can suppress epidermal cell division and maintain the balance between cell growth and apoptosis. In addition, as cAMP facilitates glycogenolysis by activating phosphorylase, the lack of cAMP can affect glycogen metabolism and lead to metabolic disorders (Ravnskjaer et al., 2016). The progressive stage of psoriasis is accompanied by a large number of inflammatory cell infiltration (Al-Harbi et al., 2020) and overactivated oxidative inflammation signals (Greb et al., 2016). Studies have shown that inhibiting oxidative inflammation signal transduction can reduce the inflammatory symptoms of psoriasis (Al-Harbi et al., 2020). The main manifestations of blood heat syndrome are related to the progressive stage of psoriasis, which may explain the abnormal expression of oxidative phosphorylation pathway in blood heat syndrome.

Furthermore, it was found that there were common differential genes between the three syndromes of psoriasis and the normal control group, which also suggests that the three syndromes, differentiated based on the dialectical perspective of TCM, have common pathogenesis. The *USP17L15* gene was significantly up-regulated in all three syndromes. This gene is related to deubiquitination, and the functions of its encoded protein have rarely been studied. However, the correlation between deubiquitination and the development and progression of skin diseases is an important direction in the current research on psoriasis (Layman and Oliver, 2016). There are numerous members in the deubiquitinase family that are important components of the ubiquitin-proteasome pathway, and can participate in skin diseases such as psoriasis and skin squamous cell carcinoma by interacting with ectodysplasin receptor, Fas-associated death domain protein, B cell-stimulating factor-3, nuclear factor kappa-B protein kinase inhibitor, and other factors (Zilberman-Rudenko et al., 2016). Therefore, USP17L15 may be involved in the pathogenesis of psoriasis (Lippens et al., 2011). *JUND* was also significantly up-regulated in all three syndromes of psoriasis, and current research shows that its encoded protein can inhibit cell apoptosis (Wang T. et al., 2015). Moreover, this gene participates in the osteoclast differentiation pathway (Beranger et al., 2007). Studies have reported that inflammatory factors of psoriasis can promote the differentiation of human monocytes to active osteoclasts, thus promoting bone injury (Raimondo et al., 2017). Therefore, it is speculated that *JUND* may be involved in the pathogenesis of psoriasis and psoriatic arthritis. As a multifunctional protein, *HNRNP* is involved in various cellular processes, including RNA splicing (Chaudhury et al., 2010), transcriptional regulation (Miau et al., 1998) and immunoglobulin gene recombination (Dempsey et al., 1999). Our results showed that compared with the normal control group, *HNRNPA2B1* was significantly down regulated in the three syndrome groups of psoriasis. Some studies have pointed out that *HNRNP* is down regulated in damaged skin of patients with psoriasis (Moldovan et al., 2019), which is consistent with our findings. This suggests that *HNRNP* is involved in the occurrence of psoriasis and may be related to the regulation of immune function related genes.

In the analysis of the common pathways between the three syndromes of psoriasis and healthy controls, it was found that there were abnormalities in the ribosome and olfactory transduction pathways in all three TCM syndromes of psoriasis. Ribosomal proteins in mitochondria participate in different cellular processes, such as cell cycle, apoptosis and mitochondrial homeostasis regulation. Mutation of *MRPS* genes in mitochondrial ribosome is related to mitochondrial dysfunction and diseases (Menezes et al., 2015; Richman et al., 2015). For example, *MRPS16* (*Bs16m*) mutation can cause mitochondrial respiratory chain disorder and abnormal ATP level (Miller et al., 2004). A study has shown that there is a correlation between cytoplasmic metabolic processes and mitochondrial ribosomes (Suhm et al., 2018). Busse et al. (2014) reported that the olfactory receptor gene *OR2AT4* is expressed in keratinocytes, and its exposure to artificial odors activates calcium signal transduction pathways, leading to wound healing. Jabbari et al. (2012), Li et al. (2014) found that the expression of *OR2T10*, *OR2T11*, *OR52B6*, *OR9Q1*, *OR10V1*, *OR1L8*, *OR2A1*, *OR2A20P*, *OR2A42*, and *OR2A9P* was down-regulated, while the expression of *OR1J1* and *ORMDL2* was up-regulated. Li et al. also found that a module closely associated with psoriasis detected by WGCNA was significantly enriched due to “olfactory receptor activity” (Li et al., 2014).

## Conclusion

In summary, the three TCM syndromes of psoriasis showed their own specific as well as some common differential genes and signaling pathways. This study organically links the three common TCM syndromes of psoriasis and provides information for clarifying the molecular basis of TCM syndromes of psoriasis.

## Data Availability

The data presented in the study are deposited in the GEO repository (https://www.ncbi.nlm.nih.gov/geo/), accession number GSE192867.
